# A new database to guide reference material selection for dietary supplement and nutrition science

**DOI:** 10.1007/s00216-024-05664-4

**Published:** 2025-01-15

**Authors:** Adam J. Kuszak, Sanem Hosbas Coskun, Stephen A. Wise

**Affiliations:** 1 Office of Dietary Supplements, National Institutes of Health, Bethesda, MD USA; 2 Kelly Government Solutions Contractor in support of the Office of Dietary Supplements, National Institutes of Health, Bethesda, MD USA; 3 ICF International Contractor in support of the Office of Dietary Supplements, National Institutes of Health, Bethesda, MD USA

**Keywords:** Dietary supplements, Foods, Natural products, Certified reference materials (CRMs), Standard reference materials (SRMs), Nutrition/nutrients

## Abstract

Rigorous research on the health effects of dietary supplements and related nutritional interventions requires thorough chemical characterization of complex matrices for their composition of macro- and micronutrients, botanical phytochemicals, and potential contaminants. Reference materials (RMs) with metrologically traceable values for these specific properties are ideal analytical tools to ensure requisite chemical measurements are reliable. However, identifying and comparing appropriate RMs for studying dietary ingredients and their metabolites is challenging, creating a barrier to reproducible regulatory testing and research. To address this challenge, the Office of Dietary Supplements at the National Institutes of Health coordinated the development of a new publicly available Reference Materials Search Tool (https://odsrmst.od.nih.gov/) database featuring comprehensive data records and search and comparison functionality. The RMST significantly expands research capacities to discover and select fit for purpose reference materials for dietary supplement and related food and natural product chemical analysis.

**Graphical Abstract:**

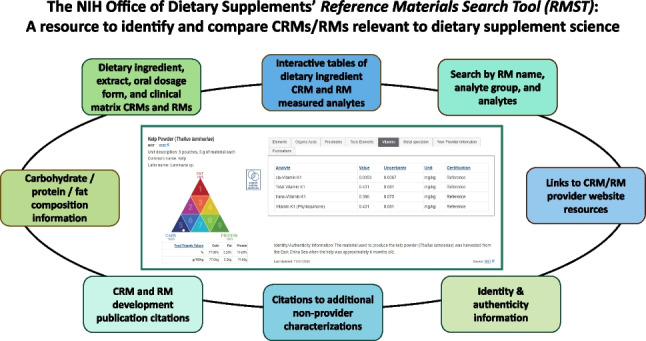

## Introduction

Dietary supplements encompass an array of nutrients and natural products including vitamins, minerals, carbohydrates, botanicals, fatty acids, proteins, and amino acids, as well as metabolites, concentrates, extracts, and combinations thereof [1]. Dietary supplement ingredients and formulations can therefore consist of vast and diverse chemical complexity. Accordingly, manufacturers must establish specifications and test for the identity, purity, strength (e.g., concentration or mass), and composition of their dietary supplement ingredients and products using scientifically valid methods [2]. Reproducible biomedical research on the health effects of dietary supplements requires that dietary interventions are replicable and that both interventions and clinical specimens reflecting nutritional status and metabolic outcomes are rigorously characterized [3].

Thorough quantitative chemical characterization is best achieved using fit for purpose standards and analytical methods formally validated for their precision, accuracy, and reliability [4]. Certified reference materials (CRMs) for dietary supplement preparations, with quantitative measurements and associated uncertainty for specific properties, are ideal tools for the development, validation, and performance assessment of methods that measure nutrients, phytochemicals, or contaminants. However, identification of appropriate CRMs and RMs can present a challenge to dietary supplement testing and research communities. Work of National Metrology Institutes (NMIs), such as the National Institute of Standards and Technology (NIST) in the USA and the National Research Council Canada, and some commercial providers have steadily increased the number of available reference materials (RMs) and CRMs relevant to dietary supplement analysis since 2000 [5, 6]. However, finding materials with value assignments for analytes of interest in matched matrices and at appropriate mass fraction levels requires searching for RM names, analytes, and manually reviewing documentation across several provider websites and collaborative databases. The Code d’Indexation des Materiaux de Reference (COMAR) [7, 8] (https://www.comar.org/) and AOAC International Technical Division on Reference Materials databases (http://tdrmdb.aoac.org/), for example, are intended to comprehensively cover RMs for all areas of research and testing (including environmental, industrial, agricultural, and clinical), and finding materials specific to dietary supplements and nutrition research requires different search strategies. Recognizing that these challenges to finding fit for purpose RMs create barriers to achieving improved industry testing and more robust dietary supplement biomedical research, the National Institutes of Health (NIH) Office of Dietary Supplements (ODS) coordinated the development of the Reference Material Search Tool (RMST) (https://odsrmst.od.nih.gov/). Featuring user-friendly search capabilities and extensive data records, the RMST can guide the selection of CRMs and RMs for dietary ingredients, supplement formulations, and their metabolites in clinical specimens. The RMST can thus support increased CRM and RM utilization in dietary ingredient and natural product chemical analyses, which should in turn promote improved rigor and reproducibility in dietary supplement science.

## RMST database purpose and scope

The ability to search for specific matrices, analyte groups, or analytes relevant to nutrition and natural product research varies across the multiple RM provider catalogs and collaborative databases. Finding and comparing the composition of multiple RMs is therefore burdensome for researchers. Additionally, a lack of text auto-fill suggestions in the search fields of existing catalogs and databases limits the ability to discover potentially fit for purpose materials when an exact matrix match is unavailable. The RMST is a specialty database designed to address challenges with identifying CRMs and RMs, as defined by the International Organization for Standardization (ISO) [9, 10], for dietary ingredients, foods, and supplements, as well as their metabolites in clinical specimens. The RMST provides robust search and compare functions for this subset of CRMs and RMs which facilitate quick assessment of a material’s fitness for a researcher’s needs. The RMST also provides expanded information for its RM records with links to publications (e.g., peer-reviewed articles, RM provider reports, or application notes) containing additional qualitative and quantitative information.

Users can find materials in the RMST through a variety of search strategies and compare multiple RMs’ composition, measured analytes, and assigned values. Users are provided links to the RM provider’s website to purchase a selected RM. The RMST records for selected CRMs and RMs are curated by NIH ODS, with a particular focus on matrix-based materials that represent dietary supplement ingredients and oral dosage forms, foods, botanicals and other natural products, and clinical specimens (e.g., plasma, serum) with values assigned for nutrition-related metabolites.

## Record content

Individual RM records in the RMST provide users with a significant amount of information on the material, primarily sourced from the publicly available Certificate of Analysis (CoA) or other reports provided by the CRM producer. With the RMST, NIH ODS also curates non-provider quantitative information and citations to publications that describe the development and certification of the materials and other significant characterization and use of the materials by researchers. RM records also have assigned tags within the RMST to increase discoverability through user searches. For example, since the term “botanical” does not occur in the CoA for Cerilliant CRM S-144 Silybum Mix Solution or the associated structured data fields in the RMST (i.e., name, source, analyte group, analyte), “botanical” is assigned as one of several tags for the record so that it will still be identified via a broader search for botanical-derived materials.

### RM provider information

**Fig. 1:**
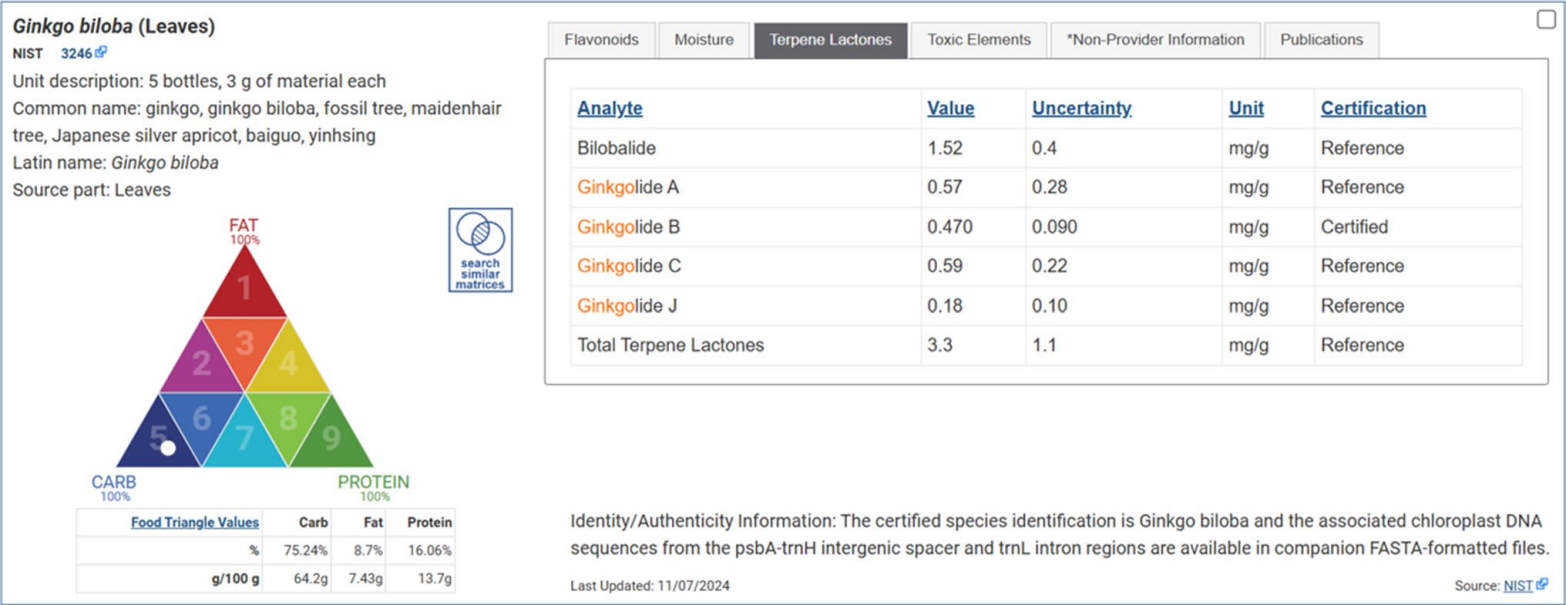
The RMST user interface record display for NIST SRM 3246 *Ginkgo biloba* (Leaves), illustrating analyte value tables, provider hyperlinks, unit descriptions, proximate composition on the Food Matrix Triangle, and material Identity/Authenticity information

Quantitative and qualitative data are arranged in a standardized format, and interactive display features facilitate comparisons of multiple RMs. As illustrated in Fig. 1, each RMST record includes comprehensive information for the material. In addition to the official name of the RM, common names, Latin binomials, and source parts are listed in the case of animal and plant derived materials, along with a description of the RM units (e.g., 3 vials, 1 mL each). The commercial provider (e.g., NIST, MilliporeSigma) is listed along with a hyperlink to the provider’s point of purchase website for more details. Most importantly, a sortable table is displayed of the specific analytes for which there are certified, reference, non-certified, or informational assigned values, with their respective uncertainty values where applicable.

Users can toggle the table’s displayed content by selecting tabs for different analyte groups, such as elements, vitamins, amino acids, alkaloids, isoflavones, curcuminoids, and fatty acids. The categorization of analyte groups is primarily derived from the nomenclature in the RM provider’s CoA, for example, the classification of NIST SRM 3247 *Ginkgo biloba* (Extract) analytes as either flavonoids, proximates, terpene lactones, or toxic elements. In some circumstances, analyte group and analyte names have been standardized for the purposes of their listing in the RMST. A material’s CoA might list either “arsenic” or “As” or either “vitamin K1” or “phylloquinone” as measurands, which are listed in the RMST as “arsenic (As)” and “vitamin K1 (phylloquinone),” respectively. Importantly, a CoA’s use of descriptive measurand terminology that indicates a particular analytical approach is reflected in RMST records. For example, distinct thyroglobulin measurements in the record for NIST SRM 1949 Frozen Human Prenatal Serum are reported as “thyroglobulin (by LC–MS/MS)” and “thyroglobulin (by immunoassay).”

Materials that consist of multiple levels or compositions are presented as multiple individual records, one for each level/composition. For example, a commercial unit of NIST SRM 3275 Omega-3 and Omega-6 Fatty Acids in Fish Oil consists of two vials each of three different oils. In RMST, SRM 3275 is organized as three separate records appended as Part 1, Part 2, and Part 3. This approach allows users to readily view and more easily discern the differences in analytes and assigned values between the RM components.

### Dietary ingredient and food RM composition

**Fig. 2:**
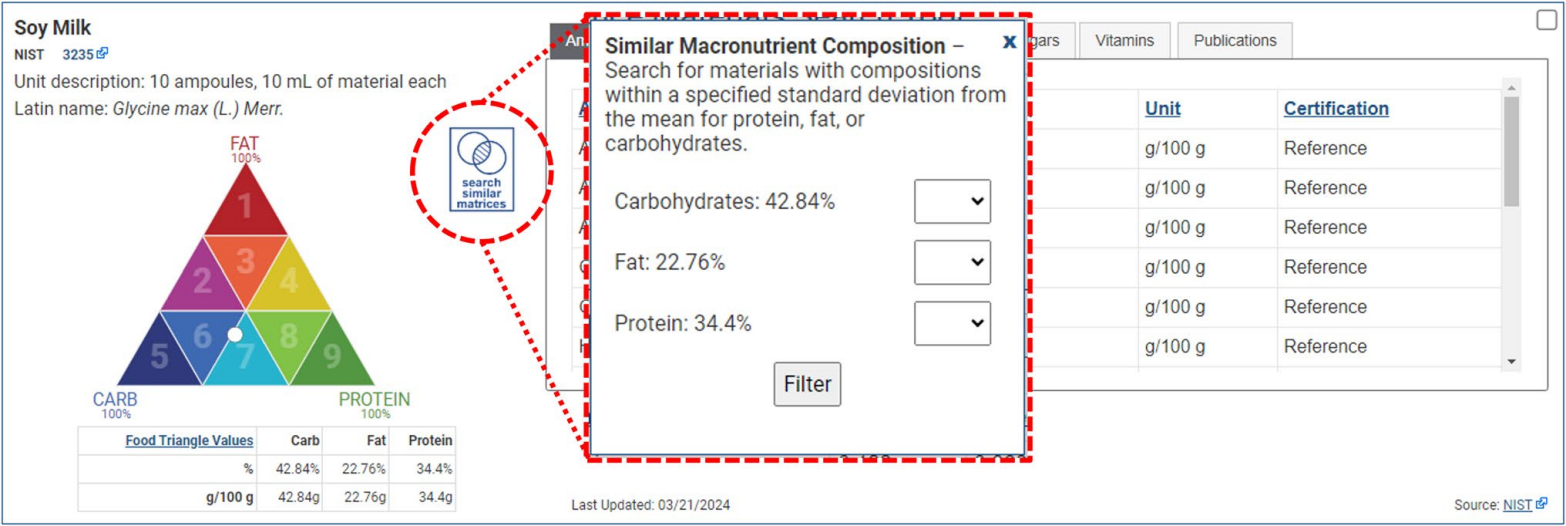
Highlight of the RMST “Search Similar Matrices” feature to identify other CRMs or RMs whose carbohydrate, protein, and fat mass fraction composition is within a set number of standard deviations from the current RM record

A well-known limitation in the use of RMs for dietary supplement and food analyses is the lack of matrix matched RMs for all ingredients and products of interest. To help users assess whether an available RM might be representative of the analytical challenges encountered in the determination of micronutrients, phytochemicals, or contaminants in their investigational ingredient or product, records for those RMs that have value determinations for total carbohydrate, total fat, and total protein will display a visual representation of the RM’s proximate composition on a model of the AOAC International “Food Matrix Triangle” previously developed to help define RMs applicable to the analysis of foods [11, 12]. This Food Matrix Triangle charts carbohydrate, fat, and protein percentages on a nine-sector triangle, where each of the main triangle’s three points represents 100% of one component and the opposite side represents 0% of that same component. An interactive “Search Similar Matrices” filter function enables users to identify other materials among the current search results that have determined macronutrient compositions within a specified standard deviation from the selected RM’s mean values for protein, fat, or carbohydrates. After clicking the “Search Similar Matrices” icon and link, users are prompted to set a filter for limiting the search to within either 0.5, 1, 1.5, 2, 2.5, or 3 standard deviations of the currently selected RM values for protein, fat, or carbohydrate (Fig. 2). These Food Matrix Triangle plots provide a means to quickly search and visualize where one RM can have a similar macronutrient composition to another RM, an important feature of the RMST that facilitates more informed comparison and selection of RMs relevant to researchers’ interests.

### Identity/authenticity information

Establishing identity and authenticity is a critical step in RM development. This is especially true for RMs intended to support the characterization of plant-based foods and botanical dietary supplements, as the source plants can exhibit significant chemical variation depending on their geographic location, environment, and harvest and preparation conditions. Where a provider’s CoA or other documentation describes unique details of an RM’s sourcing and preparation, the corresponding RMST record will display a brief text summarizing those details as Identity/Authenticity Information. The RMST record for NIST RM 8650 Ground Kudzu (*Pueraria montana* var. *lobata*) Rhizome, for example, displays text describing how the source material was harvested from Boone County, West Virginia, USA, and that taxonomic identity was established by a trained botanist. Such information is derived from the official Report of Investigation for RM 8650.

### Non-provider information

The RMST also serves as a resource for identifying chemical characterization data generated by analysts other than the RM provider, termed “Non-Provider Information” in an RM record’s data table. Non-Provider Information can include data where NIH ODS has worked with non-provider organizations to fill gaps in profiling either toxic elements or proximates for existing RMs. To date NIH ODS has collaborated with the US Food and Drug Administration (FDA) Center for Food Safety and Applied Nutrition to fill gaps in value assignments for the four major toxic elements in dietary supplement SRMs and RMs [13]. NIH ODS has also contracted with the Eurofins Nutrition Analysis Center (Des Moines, IA, USA) to measure total carbohydrate, total fat, and total protein content in botanical dietary supplement ingredient RMs that are representative of plant biomass, such as NIST SRM 3246 *Ginkgo biloba* (Leaves), SRM 3250 Saw Palmetto (*Serenoa repens*) Fruit, and SRM 3299 Ground Turmeric (*Curcuma longa* L.) Rhizome. The additional determinations for toxic elements and proximates were conducted to support more comprehensive comparisons between the available dietary supplement RMs, which researchers can use to better select appropriate control materials for method development or quality assurance efforts. Future updates of RMST records may include citations to additional non-provider analytical characterization results that are reported in peer-reviewed scientific literature, government reports, or application notes from RM producers.

### Publications

In the course of producing an RM, providers often publish reports detailing their development. The RMST provides hyperlinks to such peer-reviewed publications in scientific journals, application notes, and NMI reports in a record’s Publications tab when available. Publication hyperlinks in the RMST direct users to either a publication’s Digital Object Identifier (DOI, www.doi.org), its PubMed ID (https://pubmed.ncbi.nlm.nih.gov/), or a publisher’s URL. The primary intent of NIH ODS in curating these selected publication references is to support users in gathering more information on the material’s sourcing, preparation, value assignment, and certification process. Additional publication types may also be added to the RMST as these CRMs and RMs are further characterized in research studies, such as the metabolomic analyses by Kellogg et al. [14] of NIST SRM 3254 Green Tea (*Camellia sinensis*) Leaves and SRM 3256 Green Tea-Containing Solid Oral Dosage Form.

## Search and compare functions

RMST users can search for materials by querying keywords like “green tea,” chemical or analyte groups like “catechins,” or specific analytes like “epigallocatechin.” Running a search will query the entered term against all RM official, common, and Latin names, analyte groups, and individual analytes and alternative analyte names (e.g., thiamine and vitamin B1). A search also queries any Tags that have been applied by the RMST administration team to an RM record.

A quick search and auto-fill suggestion feature displays a drop-down menu of the various materials that might be a match for the user’s query term as it is typed in the search field. An entry of “ginger,” for example, prompts a drop-down menu listing available RM names (Ginger Extract, Ginger Rhizome, Ginger Gingerols and Shogaols Mix, etc.) as well as Analytes and Analyte Groups (gingerols, 8-gingerol, etc.). An option to add a search filter (“ + Filter” button) can be used to limit results to be inclusive of only RM records that have values for specific analyte group, analyte, or percentages of fat, carbohydrate, or protein.

**Fig. 3:**
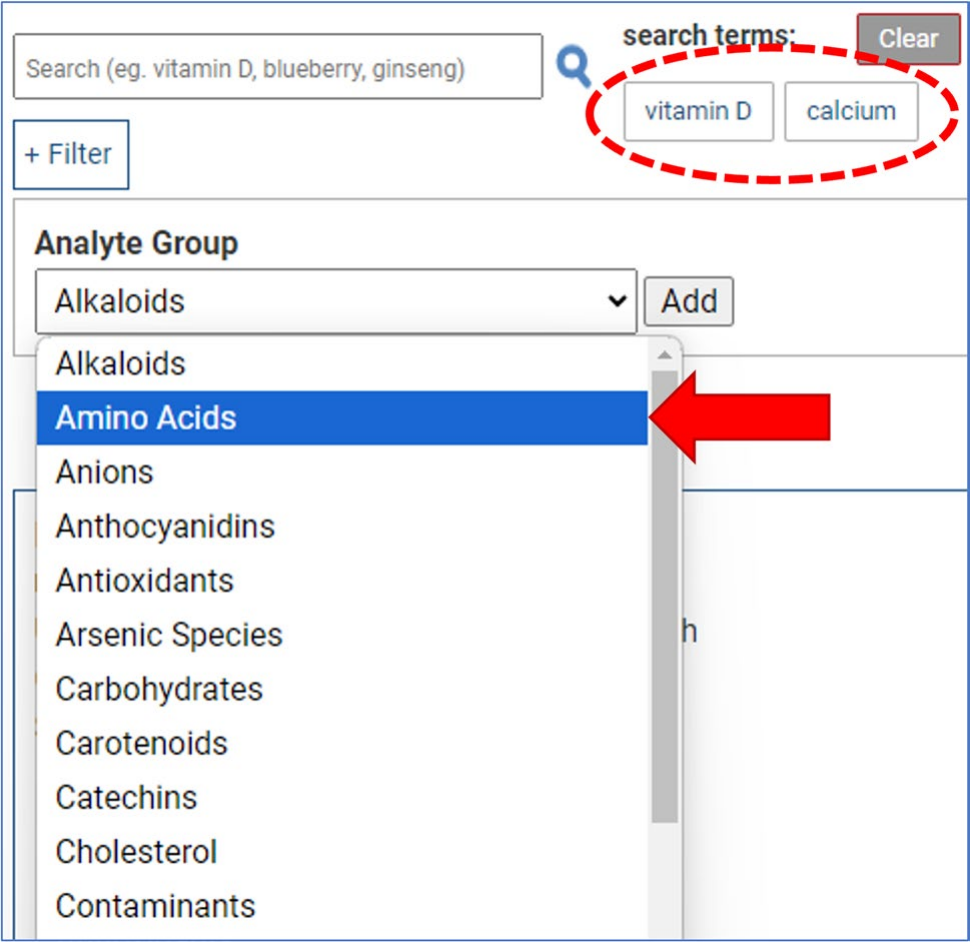
Example of the RMST search interface and filters, in which the independent queries “vitamin D” and “calcium” are displayed as search term tiles, indicated in the red circle. An added criteria, for example only returning materials with values for one or more amino acids, can be selected as an additional search filter, indicated by the red arrow

Search results are displayed as a vertical stack of record “cards” that organize all the information content for each material in a standard format. After running a search, user input text is displayed as a tile next to the search field and under a listing of search terms. Searching for additional terms will add those terms to the row of tiles, and results will be updated to be limited to only records that contain all the applied terms. For example, an initial search for “vitamin D” and a subsequent search for “calcium” will display separate tiles for each of those terms and the RMST will show only records that contain both terms (Fig. 3). Clicking an individual tile will remove that term from the search string and clicking the “Clear” button resets back to the RMST homepage. When the search term text is included in an analyte name, the corresponding analyte group tab is automatically selected in the record’s information table, and the search term text is highlighted in orange, as is seen for the search term text “ginkgo” in Fig. 1.

**Fig. 4:**
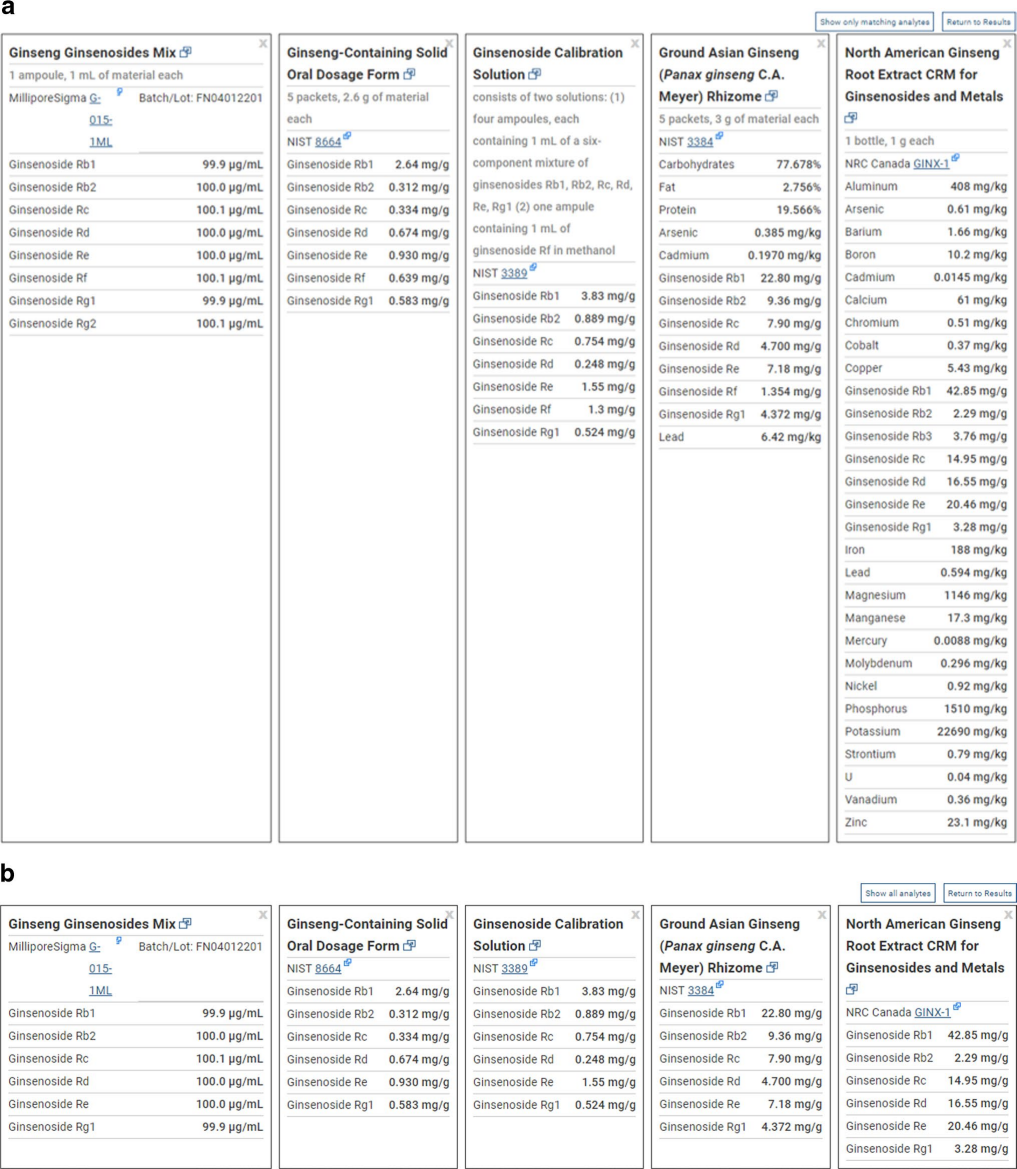
**a** Example of the RMST compare function to simultaneously view the measured analytes lists and values for 5 materials, focusing on ginsenoside values for reference materials representing a *Panax ginseng* rhizome, an extract preparation, a ginseng-containing oral dosage form, and two ginseng constituent calibration solution mixtures. **b** The same ginseng material comparison listing after limiting the display to only show matching analytes across all RMs

A key function of the RMST is a side-by-side comparison function that allows users to simultaneously view analyte and value summary tables for multiple RM records, which can help in determining which material can best suit the user’s measurement needs. Users select up to five search result materials for comparison by clicking a checkbox in the upper right-hand corner of each record card. For example, a search for “ginseng” will return eight materials, including MilliporeSigma and NIST calibration solutions, NIST oral dosage form and rhizome matrix RMs, and a NRC Canada root extract RM. Those five materials can all be viewed in the side-by-side comparison view that displays the RM’s name, unit description, provider link, and a full alphabetical listing of assigned analytes and values (Fig. 4a). Additionally, users can toggle between an option to either “Show All Analytes” or “Show Only Matching Analytes” to aid in faster, targeted comparisons between materials. In this ginseng search example, the “Show Only Matching Analytes” option will focus the user on the value ranges for the matching ginsenosides Rb1, Rb2, Rc, Rd, Re, and Rg1 (Fig. 4b).

## Record verification and maintenance

Record content for the RMST is compiled, verified, and maintained by NIH ODS. The initial data collection for RMST was conducted in 2022, in which individual RM CoA data was compiled by a primary reviewer and verified by secondary reviewers. RMST records for new RMs relevant to dietary supplement science are created either when they become commercially available, as is the case where NIH ODS has partnered with the RM provider, or when the new relevant RM is identified by NIH ODS during twice-yearly data collection activities. An RM’s CoA may be revised over time to report new, updated, or removed values, and in these instances, an RMST record will be updated to reflect such revisions. Existing records are scheduled to be reviewed twice yearly. A time stamp for each record indicates the date of its latest update, and users should always confirm an RM’s composition, value assignments, and availability by following the hyperlink to its provider’s website resources.

In the interest of helping users understand the history and evolution of these dietary supplement and nutrition related RMs, the RMST contains archival records for materials that have been replaced or discontinued. Records for discontinued materials, such as NIST 3240 *Ephedra sinica* Stapf Aerial Parts, prominently display the text “*Discontinued” next to the RM name. The availability of such archival information in the RMST serves as a resource for researchers when reviewing past publications that report using discontinued RMs without detailing their composition.

## Accessibility

All RMST content, functionality, and documentation are publicly accessible and free of charge at https://odsrmst.od.nih.gov/. Public feedback on the RMST is welcomed, especially in the interest of improving and expanding functionality for users and ensuring that the information contained within is complete, accurate, searchable, and accessible. Users can share their questions and comments with RMST managers via email at ODS.AMRM@nih.gov.

## Conclusions and future directions

Through the curated Reference Materials Search Tool, NIH ODS has set out to make the identification and selection of fit for purpose reference materials for dietary supplement characterization and related nutrition investigations easier for the analytical and research communities. With RMST, scientists can discover and compare the properties of CRMs and RMs that represent the types of analytical challenges often encountered when measuring vitamins, minerals, phytochemicals, fatty acids, and other bioactive or marker constituents in the complex matrices that are dietary ingredients, foods, and dietary supplements. Key novel features of the RMST that significantly expand its utility beyond a collection of RMs relevant to dietary supplement and nutrition analysis are (1) its use of the Food Matrix Triangle to visualize proximate composition similarity among materials, (2) the ability to simultaneously compare the analytes and mass fraction ranges across multiple materials, (3) the inclusion of non-provider analytical characterization data, and (4) a listing of citations to RM development, certification, and additional characterization publications.

Information for 120 dietary supplement relevant CRMs and RMs is currently reported in the RMST, covering over 45 analyte groups and approximately 475 measurands of interest, and that number will continue to grow as new materials become available. Likewise, peer-reviewed publication citations and providers’ reports and additional RM characterizations will be added on a continual basis to disseminate the results of characterization studies that increase knowledge on dietary supplement CRM and RM composition. Additional search features are in development that refine capabilities to compare the composition of proximates for plant- and food-based RMs, which will improve a user’s ability to decide whether an RM that is not an exact matrix match for their sample can still prove useful in determining method performance. Importantly, efforts are underway to expand RMST coverage of clinical laboratory CRMs and RMs, meaning serum, plasma, or urine matrices that have value assignments for various vitamin isomers, fatty acids, and other dietary metabolites of interest to researchers. This inclusion of clinical materials in RMST supports researchers in finding RMs to both verify the chemical composition of their experimental dietary interventions and measure the intervention’s intake or health effect by assessing metabolites in clinical specimens.

## Declarations

### Disclaimer

The opinions expressed in this article are the authors’ own and do not reflect the view of the National Institutes of Health or the US government. Any citations of commercial organizations or trade names in this report do not constitute an official NIH or US government endorsement or approval of the products or services of these organizations.
